# Advancing Research on Productive Aging Activities in Greater Chinese Societies

**DOI:** 10.1007/s12126-012-9171-2

**Published:** 2012-10-07

**Authors:** Terry Yat-sang Lum

**Affiliations:** Sau Po Centre on Ageing and Department of Social Work and Social Administration, The University of Hong Kong, Pokfulam, Hong Kong

**Keywords:** Productive aging, Productive aging research, Review on productive aging, Productive aging in China

## Abstract

The public discourse on productive aging as a research and policy initiative has just begun in greater China. Two conferences in Mainland China in 2009 and 2011 and subsequent conferences in Taiwan and Hong Kong in 2012 have set it in motion. Because applied social science research has just started in greater China, researchers in Chinese societies will benefit from the experience and rich literature accumulated over the last three decades in the West. In this paper, I review and reflect on the research methods used in productive aging research in both Chinese societies and in the West. I believe that to advance productive aging research in greater China, we need to (1) discuss and agree upon a definition of productive aging, (2) identify and differentiate outputs and outcomes of productive aging activities in greater China, (3) develop precise measures for productive aging involvement, (4) focus on institutional (program and public policy) factors that promote productive aging involvement, (5) use a strong research design (such as a quasi-experimental design) to establish the internal validity of productive aging programs, and (6) be theory-driven. Lastly, productive aging should be seen as a choice, not an obligation for older people; otherwise, the productive aging agenda will be seen as exploiting older people. It is important that Chinese researchers and policy-makers have this in mind when they are advocating productive engagement of older people in China.

“Productive aging” refers to older people’s participation in various activities that contribute to generate goods or services or develop the capacity to produce goods or services (Aquino et al. 1996; Sherraden et al. 2001; Lo Sasso and Johnson 2002). These include activities such as full- or part-time employment, formal and informal volunteering, grandparenting, and caregiving for frail family members and friends. Productive engagement of older people is not new in the history of humankind (Achenbaum 2001). In fact, retirement and other forms of formal disengagement in productive activities are relatively new practices in industrial society; in agricultural societies, most older people are continuously involved in productive activities in family farms throughout their life cycle (Windsor et al. 2008). In China, the concept of productive engagement of older people was documented more than 2,500 years ago (Song et al. 2010). It is considered a virtue in Chinese culture to provide older people with opportunities to contribute to their families and societies. The concept of productive aging was introduced to Chinese communities in a series of conferences in Shandong (2009) and Beijing (2011) in Mainland China, as well as in Taipei (2012) and Hong Kong (2012). The first Chinese book on productive aging was published after the first conference in Shandong.

The term “productive aging” in the West was first used by Robert Butler in 1982 to advocate for a change from the negative perception of elderly dependency at the time (Butler and Gleason 1985). During the last three decades, public discourse on productive aging has gradually shifted from advocating the productive aging agenda to scientific research documenting the rate, type, and intensity of productive aging activities; identifying factors affecting participation in productive aging activities (Hinterlong et al. 2001; McNamara and Gonzales 2011); and, more recently, demonstrating the effects of these activities on the well-being of older people (Musick et al. 1999; Morrow-Howell et al. 2003; Lum and Lightfoot 2005). The bulk of the research on productive aging in the West has focused on older people volunteering because of the compelling idea that volunteering improves the lives of older people and strengthens civil society (Morrow-Howell 2010). In recent years, researchers have started trying to find the optimal level of productive aging activities for the well-being of older people and the mechanism leading to the positive effects of productive aging activities (Tang and Morrow-Howell 2008; Tang et al. 2009, 2010).

In Chinese societies, productive aging is an emerging concept and practice. The discussion of older people’s role in society has so far focused on positive aging and successful aging (hereafter, we use the term “successful aging” to represent both concepts). These two terms are often interchangeable and have only been loosely defined to include civic engagement such as volunteering, a healthy lifestyle, good psychosocial adjustment in old age, financial security, and good health (House 2010). They are more inclusive concepts than the concept of productive aging we use in this paper. Such a broad definition makes it difficult or even impossible to develop a unified measure of successful aging or evaluate policy and program options that promote successful aging.

There exist a few comprehensive reviews on productive aging research in the West; the goal of this paper is not to repeat those efforts (Lum and Lightfoot 2005; Morrow-Howell 2010); instead, this paper reviews and reflects on the research methods used in that research, with specific suggestions on ways to advance the agenda of productive aging research in greater Chinese societies.

## Research Methods in Productive Aging Studies in the West

The last three decades have witnessed a rapid development of applied social science research methodology. The information technology revolution has allowed social scientists to use advanced multivariate statistics on personal computers, significantly reducing the cost of such analysis. In addition, more large nationally representative survey datasets are available for applied social science research, such as the Americans’ Changing Lives Study (House 2010) and the Health and Retirement Study (Juster and Suzman 1995). A few of these datasets are longitudinal, such as the Health and Retirement Study, allowing researchers to investigate the trajectory of productive aging activities and the impacts of productive aging activities on the well-being of older people as they age. Research on productive aging has benefited from these developments and advanced gradually over the last three decades.

In the first decade after the concept was introduced, productive aging studies were mostly descriptive, focusing primarily on documenting the trends of volunteering and employment of older people, the demographics of older volunteers and workers, and the factors affecting their motivations to work or to volunteer (Rouse and Clawson 1992; Chambré 1993; Kuehne and Sears 1993; Hayward et al. 1994; Herz 1995; Aquino et al. 1996; Rix 1996; Lum and Lightfoot 2005). Early studies also documented the rate, type, and intensity of various productive aging activities.

In the second and third decades, studies gradually became more analytical and used more data from large national surveys (Musick et al. 1999; Luoh and Herzog 2002; Morrow-Howell et al. 2003; Lum and Lightfoot 2005; Ozawa and Lum 2005; McNamara and Gonzales 2011). Guided by a few social gerontology theories, these studies tried to identify factors that affected older people’s participation in productive aging activities, and to document the benefits productive aging activities have on older people’s physical health, mental health, and well-being.

During the third decade, two trends emerged in productive aging research. The first trend focused on uncovering institutional factors, such as organizational support(Tang et al. 2010), compensation, and recognition (Tang et al. 2009) and volunteer management practices(Hager 2004), that affected older people’s participation; the second trend focused on testing specific programs that promoted the productive engagement of older people (Tang and Morrow-Howell 2008; Butrica et al. 2009; Hong et al. 2009; Tang et al. 2009, 2010). These studies also became very analytical, using advanced statistical techniques to test specific social theories. In recent years, the focus has gradually shifted to intervention research in order to identify the optimal level of productive aging activities to maximize the benefits to older people and their communities (Fried et al. 2004; Carlson et al. 2008; Morrow-Howell et al. 2009; Hong and Morrow-Howell 2010). Research designs have shifted from cross-sectional to longitudinal and to quasi-experimental or even experimental with purposely recruited participants.

In China, research on productive aging is still at the early phase of development. Researchers have started to document the prevalence of productive aging activities of older people (Kawn 2000; Mjelde-Mossey and Chi 2005; Wu et al. 2005; Schwingel et al. 2009; Chong 2010). The development of productive aging research in Chinese society will benefit from experience accumulated over the last 30 years in the West. As suggested by Morrow-Howell and colleagues (2001), research should move from the descriptive to the analytic and evaluative. They have advocated using well-specified theory to explore social and organizational structures associated with productive aging and to conduct demonstration programs to test the actualization of productive potential (Sherraden et al. 2001).

## Advancing Research on Productive Aging Activities in Chinese Societies

As discussed above, the concept of productive aging is not new to China. However, productive aging as a research topic and as a public policy agenda is new. Chinese scholars have not yet vigorously debated the definition of the concept of productive aging. As a result, there is no consensus on which activities should be included and which should not. In the first productive aging conference in China in 2009, “productive aging” was translated as “ 产出性老龄化,” which focused more on the market-based economic activities of older people. Some scholars believe that this Chinese term is too narrowly defined. Also, in Mainland China, because of the high unemployment rate of young people, there is a rigid and compulsory retirement age for all workers in urban areas. Employment opportunities for older people in the city are very limited. The market-based focus of the Chinese term may significantly limit the development of productive aging public policy in China. At the second productive aging conference in Beijing in 2011, the term was translated as “ 老有所為.” This term focuses on a broader productive engagement of older people, allowing scholars to include many non-market-based activities that still have economic value.

To advance productive aging as a research agenda and policy initiative in greater Chinese societies, it is important for Chinese scholars to develop a consensus on the definition of productive aging in a Chinese context. In other words, they need to decide which activities should be counted as productive aging activities. In particular, I suggest that Chinese scholars use the four categories suggested by Sherraden and his colleague as a starting point and then work out, under each category, the productive aging activities that fit Chinese societies and culture. These four categories are market activities (such as employment), non-market activities with economic value (such as taking care of older family members or young children), formal social and civic activities (such as volunteering in schools and social services agencies), and informal social assistance (such as helping neighbors) (Sherraden et al. 2001). This will allow researchers to clearly differentiate the concept of productive aging from other conceptually related terms, such as successful aging. This will also make it easy for Chinese scholars to agree upon a set of outcome and output measures for the productive engagement of older people.

Second, it is important for Chinese scholars to differentiate the outputs and outcomes of productive aging activities. The primary output of productive aging is the quantity of goods and services older people produce (such as volunteer hours and number of people served). The primary outcome of productive aging activities is the economic value of these goods and services. Economic value is a vague concept and can only be measured when there is a market for the transaction of such goods and products. For example, the price of a work of art may be millions of dollars in good economic times, but much less during economic recession or wartime. Similarly, a glass of clean drinking water may be worth very little in most urban cities, but much higher in a desert.

Many Western researchers have used the concept of opportunity costs to quantify the economic value of productive aging activities; that is, if these goods and services were not produced by older people, how much money would be needed to purchase the same quantity and quality of goods or services from the marketplace? For example, the economic value of volunteer hours can be measured by the costs of hiring qualified staff in the labor market to produce the same amount of staff hours. Similarly, the economic value of caring for a frail family member can be measured by the cost of hiring a personal care assistant from a formal service provider to perform the same amount of care. The ability to assign an economic value to specific productive aging activities will allow Chinese scholars to monetize and compare the relative contribution of these productive aging activities in Chinese societies.

It is also important for Chinese scholars to pay attention to the secondary outcomes (or benefits) of productive aging activities. I use the term “secondary outcome” to refer to non-good and non-service benefits of productive aging activities, such as better physical health and mental health outcomes, better adjustment in later life, and higher life satisfaction for people who participate in productive aging activities. In Western research, it is well documented that volunteering enhances older people’s physical and mental health (Musick et al. 1999; Morrow-Howell et al. 2003; Lum and Lightfoot 2005). Although it is very difficult to come up with a standardized approach to assign an economic value to the improved physical and mental health status, we will still be able compare these benefits across different productive aging activities if we have valid and reliable measures for these benefits. Many of these measures are available in Western countries and in Chinese societies outside Mainland China, such as Taiwan and Hong Kong (e.g. the geriatric depression scale, self-rated health, activities of daily living, life satisfaction and SF-36). However, those measures will not be directly applicable to Mainland China without re-validation. It is therefore essential that Chinese scholars systematically develop and validate a set of standardized health and psychosocial measures to quantify these secondary outcomes.

Third, while the above discussion has focused on measuring output and outcome, it is equally important to be able to precisely measure productive aging input. Many early researchers used imprecise measures of productive aging activities, such as a dichotomous variable to measure whether older people were involved in productive activities. In order to advance the productive aging field in Chinese societies, researchers need to be able to measure the type, form, intensity, and duration of these productive aging activities precisely. Similarly, information about other program characteristics also needs to be collected.

So far, most productive aging research in Chinese societies has used a cross-sectional design (Mjelde-Mossey and Chi 2005; Wu et al. 2005; Chong 2010). Cross-sectional designs are good to develop descriptive and, perhaps, explanatory knowledge. However, we need to develop the capacity to use quasi-experimental or even experimental designs to produce knowledge that can be used to develop programs and policies to promote productive aging activities or to evaluate the relative effectiveness of different productive aging activities. Both designs involve the use of at least one comparison group and data collection over time—before, during, and after the intervention. These designs will allow researchers to eliminate most threats to internal validity and demonstrate stronger associations between interventions and outcomes. When doing intervention research on program effectiveness, it is important that researchers first focus on establishing internal validity—rather than external validity—of the targeted productive aging activity. To test internal validity, researchers need to use the maximum dosage of their intervention and try to find the maximum effect. This will allow the establishment of a causal relationship between interventions and outcomes. Once the effectiveness of an intervention is established, the logical next step is to find out the minimum intervention dosage needed to sustain the effect of the intervention. Once the effect of a specific productive aging activity is documented in a sample, researchers should manualize the intervention and increase the external validity by replicating the intervention at different sites.

Finally, social theories that explain the linkage between productive aging activities and their outcomes were developed in the West (such as the role theory and the integrated sociology theory proposed by Wilson and Musick (Wilson and Musick 1997)). While it is likely that these theories will still be valid in Chinese societies, this is an empirical research question that needs to be tested. It is important that Chinese scholars be theory-driven when designing their productive aging programs and research. It is equally important that Chinese researchers test the validity of productive aging theories developed in the West in Chinese contexts. Greater China is a huge geographic area with much diversity in language, culture, and social and economic development. Researchers also need to pay attention to the diversity within China when testing these theories.

## Conclusion

The public discourse on productive aging as a research and policy initiative has just begun in greater China. The two conferences in Mainland China in 2009 and 2011 and subsequent conferences in Taiwan and Hong Kong in 2012 have set the discourse in motion. Because applied social science research has just started in greater China, researchers in Chinese societies will benefit from the experience and rich literature that the West has accumulated over the last three decades. In this paper, I have highlighted some research method issues of productive aging research. I believe that, to advance productive aging research in greater China, we need to (1) discuss and agree upon a definition of productive aging, (2) identify and differentiate outputs and outcomes of productive aging activities in greater China, (3) develop precise measures for productive aging involvement, (4) focus on institutional (program and public policy) factors that promote productive aging involvement, (5) use strong research designs (such as quasi-experimental designs) to establish the internal validity of productive aging programs, and (6) be theory-driven. Lastly, productive aging should be seen as a choice, not an obligation for older people. Otherwise, the productive aging agenda will be seen as exploiting older people (Hinterlong et al. 2001). It is important that Chinese researchers and policy-makers have this in mind when they advocate productive engagement of older people in China.
